# Gene loss and genome rearrangement in the plastids of five Hemiparasites in the family Orobanchaceae

**DOI:** 10.1186/s12870-018-1249-x

**Published:** 2018-02-06

**Authors:** Daniel C. Frailey, Srinivasa R. Chaluvadi, Justin N. Vaughn, Caroline G. Coatney, Jeffrey L. Bennetzen

**Affiliations:** Department of Genetics, University of Georgia, Athens, GA 30677 USA

**Keywords:** Chloroplast, Chromosome rearrangement, Gene deletion, Parasite, Plastome, *Striga*

## Abstract

**Background:**

The chloroplast genomes (plastome) of most plants are highly conserved in structure, gene content, and gene order. Parasitic plants, including those that are fully photosynthetic, often contain plastome rearrangements. These most notably include gene deletions that result in a smaller plastome size. The nature of gene loss and genome structural rearrangement has been investigated in several parasitic plants, but their timing and contributions to the adaptation of these parasites requires further investigation, especially among the under-studied hemi-parasites.

**Results:**

De novo sequencing, assembly and annotation of the chloroplast genomes of five photosynthetic parasites from the family Orobanchaceae were employed to investigate plastome dynamics. Four had major structural rearrangements, including gene duplications and gene losses, that differentiated the taxa. The facultative parasite *Aureolaria virginica* had the most similar genome content to its close non-parasitic relative, *Lindenbergia philippensis*, with similar genome size and organization, and no differences in gene content. In contrast, the facultative parasite *Buchnera americana* and three obligate parasites in the genus *Striga* all had enlargements of their plastomes, primarily caused by expansion within the large inverted repeats (IRs) that are a standard plastome feature. Some of these IR increases were shared by multiple investigated species, but others were unique to particular lineages. Gene deletions and pseudogenization were also both shared and lineage-specific, with particularly frequent and independent loss of the *ndh* genes involved in electron recycling.

**Conclusions:**

Five new plastid genomes were fully assembled and compared. The results indicate that plastome instability is common in parasitic plants, even those that retain the need to perform essential plastid functions like photosynthesis. Gene losses were slow and not identical across taxa, suggesting that different lineages had different uses or needs for some of their plastome gene content, including genes involved in some aspects of photosynthesis. Recent repeat region extensions, some unique to terminal species branches, were observed after the divergence of the *Buchnera/Striga* clade, suggesting that this otherwise rare event has some special value in this lineage.

## Background

Beyond its essential roles in photosynthesis, the chloroplast is also the specialized site for synthesis of some pigments, lipids, amino acids, and sulfur compounds [1, 2]. The chloroplast contains its own genome, the plastome. The plastome in vascular plants is believed to have a single endocytotic origin, more than a billion years ago, from a cyanobacterium-like organism [3–5]. Over time, most genes of the cyanobacterium have either been lost or have migrated to the nuclear and/or mitochondrial genomes, so that modern plant plastomes contain approximately 10% of the ancestral endosymbiont genes [6].

Plastomes are under strong selective pressure in most plants and therefore tend to be highly conserved in terms of gene content and order, nucleotide substitution rates, structure, and size [7]. Most plastomes range between 120 to 160 kb in size [1]. Originally, plastomes were thought to be arranged as single circular molecules. Although a percent of plastomes do have this structure, they are also present in dimers and higher order concatemers, in both circular and linear configurations [2]. Plastomes have a quadripartite structure consisting of a large single copy (LSC) region and a small single copy region (SSC) separated by two virtually identical inverted repeat (IR_A_ and IR_B_) regions [8]. Recombination between the two inverted repeats plays a role in stabilizing the plastome [9]. In angiosperms, LSCs are typically 80–90 kb, SSCs 16–27 kb, and inverted repeats 20–30 kb [2]. The plastome contains genes encoding proteins involved in photosynthesis as well as the other biochemical pathways carried out in the chloroplast. It also encodes 30 tRNAs and several other structural RNAs [10–12].

Unlike most species of plants, parasitic plants often contain highly divergent plastomes [13–28]. Non-photosynthetic plants that obtain all their nutrients from the host plant are called holoparasites, while hemiparasites are photosynthetic plants that obtain some nutrients from the host and the rest from photosynthesis. Hemiparasites can be further broken down into obligate parasites that require a host plant during at least part of their life cycle or facultative parasites that are capable of completing their entire life cycle without a host plant, but can parasitize if a host is available. Parasitic plant plastomes can have lower selective pressure due to less reliance on photosynthesis. Not surprisingly, holoparasites have the potential to lose all of their photosynthetic genes, or perhaps the entire chloroplast genome [24], while hemiparasites are expected to maintain most or all of their photosynthetic genes. Lack of or less selection for retained photosynthetsis and other vital chloroplast functions can allow higher nucleotide substitution rates and rearrangements in the plastome. These changes can include functional gene loss through pseudogenization or physical loss through gene deletion, resulting in a smaller plastome size [14, 16, 17, 19–23, 26]. In some cases, an entire copy of one of the inverted repeats has been lost [19, 20, 29]. Since the repeats play a stabilizing role in plastome structure, this leads to further destabilization and rearrangements in the plastome [30].

The Orobanchaceae are the largest family of parasitic plants. They contain a single non-parasitic genus, *Lindenbergia* [31], in addition to holoparasites and both facultative and obligate hemiparasites [32]. In this study, we looked at two species of facultative hemiparasites, *Aureolaria virginica* and *Buchnera americana,* and three species of obligate hemiparasites all in the genus *Striga*: *S. hermonthica*, *S. forbessii*, and *S. aspera*. The plastome of the autotroph *Lindenbergia philippensis* had already been fully sequenced and annotated by Wicke et al. 2013, so it provided a valuable outgroup to compare to our parasitic species. Despite the fact that all of our studied species rely on photosynthesis, we discovered numerous cases of gene loss and other forms of rearrangement in their plastomes.

## Methods

### Sample collection, DNA extraction and DNA sequencing

DNA preparations from leaf tissue of *Striga* species were the same as those previously described [33]. *B. americana* and *A. virginica* leaf tissues were collected from live specimens in the Plant Biology Teaching collection at the University of Georgia. High molecular weight DNAs were isolated from these samples by a modified cetyltrimethylammonium bromide procedure [34] and were used for Illumina library preparation with the NEBNext® Ultra™ II DNA Library Prep Kit as per manufacturer’s instructions. Illumina MiSeq PE250 runs were performed at the Georgia Genomics Facility.

### Plastome assembly

Plastid-homologous sequences were selected from the total cellular DNA reads of *Striga hermonthica* and then assembled with a de novo assembly pipeline, both as previously described [35], to create ten contigs. To fill the ten gaps in this assembly, primers were designed for the end of each contig and PCR was set up for each primer pair. Each PCR reaction consisted of 5–20 ng template DNA, 10 μL Q5 Reaction Buffer, 10 μL betaine, 2.5 μL DMSO, 1 μL 10 mM dNTPs, 2.5 μL forward primer, 2.5 μL reverse primer, .5 μL Q5 High-Fidelity DNA Polymerase, and 20 μL water. Touchdown PCR was performed on each reaction on an MJ Research PTC-200 Peltier Thermal Cycler. The program used was 98 °C for 30s; 10 cycles consisting of denaturation at 98 °C for 20 s, annealing at 68 °C - 1 °C each cycle for 15 s, elongation at 72 °C for 1 min; 25 cycles consisting of denaturation at 98 °C for 15 s, 58 °C for 15 s, elongation at 72 °C for 1 min; and a final elongation step at 72 °C for 10 min. PCR products were sent to Macrogen USA (Rockville, MD) for Sanger sequencing. Sequences were manually assembled to the original contig ends in order to fill gaps.

The assembled *S. hermonthica* plastome was aligned to the *Lindenbergia philippensis* plastome using Mauve 2.3.1 [36]. All junctions to predicted rearrangements in the *S. hermonthica* plastome were PCR amplified and Sanger sequenced (Macrogen USA) to confirm that the assembly was correct.

Plastome reads for each of our target species were extracted from raw total DNA reads by identifying reads homologous to our S. hermonthica assembly and used in de novo assemblies with Geneious 8.1.6 [37]. Hence, each de novo assembly was performed independently for each species.

The repeat region of each assembly was identified by the region of the plastome having approximately two times the read coverage compared to the rest of the plastome. In the case of *S. hermonthica*, we ran PCR on all the junctions between the repeats and the LSC and SSC. Sequencing of these PCR products confirmed the assembly of these junctions.

### Plastome annotation and alignment

The genes from the annotated *L. philippenis* plastome were downloaded from NCBI along with the plastome genes from all plant species from Chloroplast Genome DB [38]. We ran BLAST on all genes against each assembled plastome. All genes identified in the plastome were then mapped to the plastome using Geneious. Reads were also mapped to each gene individually to confirm that the sequence assembly was correct and that there were no genes missing in our assemblies that had reads map to them. Individual genes were aligned across all five parasitic species as well as *L. philippensis* using Clustal W [39] to identify mutations, insertions, and deletions. Genes that contained one or more frameshift mutations or premature stop codons were considered potential pseudogenes. The raw reads for the most 5′ predicted stop codons or frameshift mutations that could have created a pseudogene were visually inspected to evaluate the accuracy of the read call. Read depth varied from 34X to 548X across these sites, with 18 cases giving 100% agreement with the mutational call, and the others showing > 95% agreement (data not shown).

The plastomes were aligned using Mauve 2.3.1. The phylogenetic tree was generated with sequence data from all intact genes shared by all five species. These genes were concatenated into a single sequence, which were aligned using Clustal W in Mega 7.02 [40]. *L. philippensis* was included as an outgroup. Bayesian phylogenetic analyses were done on MrBayes 3.2.1 [41] using Generalized Time Reversible + Γ + I model of evolution. We ran an initial 2.0 × 10^7^ trees with sampling every 10,000 generations. The first 5.0 × 10^6^ trees were not included in the final analysis. The remaining trees were used to generate a consensus tree. The same data were used to generate a phylogenetic tree using MEGA [40], and this generated nearly identical results, so only the MrBayes tree is presented.

## Results

### Plastome assembly

Illumina sequence analysis of total DNA from leaves provided sufficient plastome-homologous sequence data for full genome assembly of all five studied species. All of the initial contiguous sequences were confirmed in their order, and gaps filled, by PCR and Sanger sequencing of the PCR product. Hence, complete assemblies were obtained, and any predicted rearrangements by comparisons of these species genomes were also confirmed by PCR and Sanger sequencing.

Our assemblies indicated that the plastome of *A. virginica* is 153,547 bp, close in size to the 155,103 bp plastome of *L. philippensis.* The lengths of the LSC, SSC, and IR (84,317 bp, 17,168 bp, and 26,031 bp, respectively) in *A. virginica* were all similar in size to those in *L. philippensis* (85,584 bp, 17,885 bp, and 25,812 bp, respectively). The plastomes from the other four species (*S. hermonthica, S. aspera*, S. *forbessii and B. americana)* showed major differences in both total plastome size and the size of each region*.*

### Repeat expansion

The three *Striga* species and *B. americana* all exhibited larger plastome sizes, ranging from 166,596 bp in *B. americana* to 190,233 bp in *S. forbessii*. The larger plastome sizes correlate with the larger IR lengths, ranging from 43,864 bp in *B. americana* to 63,240 bp in *S. forbessii*. Conversely, the LSC and SSC regions in all four species showed a reduction in size. SSC lengths ranged from 3377 bp in *Buchnera* to 11,191 bp in *S. forbessii*, while the LSC lengths ranged from 51,628 bp in *S. hermonthica* to 75,491 bp in *B. americana.* All plastome component sizes are shown in Table 1, and are compared to those for some previously studied parasitic plants.

**Table 1:** Plastome composition in *Lindenbergia philippensis* and eight hemiparasites of the Orobanchaceae

	Plastome Length (bp)	LSC Length (bp)	SSC Length (bp)	IR Length (bp)	Expansion of IR (bp)	IR from LSC (bp)	IR from SSC (bp)	IR Genes from LSC	IR Genes from SSC
*L. philippensis*	155,103	85,606	17,885	25,800	NA	NA	NA	NA	NA
*S. americana*	160,910	84,756	6517	34,818	9018	0	6334	0	2
*P. cheilanthifolia*	155,159	85,256	17,971	25,966	0	0	0	0	0
*A. virginica*	153,547	84,317	17,168	26,031	0	0	0	0	0
*T. versicolor*	152,448	83,650	17,520	25,639	0	0	0	0	0
*B. americana*	166,596	75,491	3377	43,864	18,744	8050	10,694	7	8
*S. forbessii*	190,233	52,563	11,191	63,240	37,218	32,681	4536	34	1
*S. hermonthica*	186,418	51,628	9884	62,453	36,799	32,299	4500	34	1
*S. aspera*	185,932	51,706	10,504	61,861	36,201	31,986	4215	34	1

The table shows the size of the IR, how much the IR has expanded compared to *L. philippensis*, the amount of the expanded repeat sequence that came from the LSC and from the SSC, the number of originally single-copy genes now found in the IR, and the number of genes originally found in the LSC and SSC now contained in the IR. The *Aureolaria*, *Buchnera*, and *Striga* results are from our analysis while the other data were previously published for *Lindenbergia philippensis*, *Schwalbea americana* [19], *Pedicularis cheilanthifolia* [28], *Triphysaria versicolor* [20]

In *B. americana*, repeat expansion has occurred in both the LSC and SSC regions. Approximately 8 kb of LSC sequence flanking the repeat in *L. philippensis* is now included in the repeat region in *B. americana*. This region contains the genes *trnH*, *psbA*, *matK*, *trnK*, *rps16*, *trnQ*, and *psbK*. The expansion also occurred into both flanking regions of the SSC. On one end of the SSC, *ndhF* has been lost in *B. americana* while the adjacent 6 kb region is now part of the repeat region*. ndhE*, *psaC*, *ndhD*, *ccsA*, *trnL*, and *rpl32* are all contained within this region. On the other end of the SSC, an approximately 5 kb region containing *rpl32* and *ycf1* has been shifted into the repeat in *B. americana*.

The three *Striga* species show an almost identical pattern of IR expansion, with the majority of expansion occurring in the LSC region. The two LSC-flanking regions as well as an internal region are now part of the repeat in all three *Striga* species. An approximately 12.5 kb region flanking the IR in *L. philippensis* is part of the IR in *Striga.* This region contains trn*H, psbA, matK, trnK, rps16, trnQ, psbK, psbI, trnS, trnG, trnR,* and *atpA*. On the other end of the LSC, the IR region has expanded into approximately 18.8 kb of adjacent sequence. This region contains 15 genes: *clpP, psbB, psbT, psbN, psbH, rpoA, rps11, rpl36, infA, rps8, rpl14, rpl16, rps3, rpl22*, and *rps19*. The internal region is 5.5 kb and contains *ycf4, cemA, petA, psbJ, psbL, psbF*, and *psbE*. In *L. philippensis,* there are 61 kb between this region and the IR on one side and 18 kb between this region and the IR on the other side. Approximately 4.5 kb of the SSC region adjacent to the repeat is now part of the repeat region in all three *Striga* species. This region contains the *ycf1* gene. All details of repeat expansion are shown in Table 1.

### Overall gene content

No tRNA or rRNA genes have been lost in any of the five species. All of the plastomes encode 30 different tRNAs and 4 different rRNAs. Compared to the outgroup *Lindenbergia*, *Aureolaria* has not lost any protein-coding genes and contains a total of 79 single copy genes and six more that are contained within the repeat region and therefore present in two copies. *Buchnera* has a total of 77 single copy genes, plus 17 in the repeat regions, with 9 genes being possible pseudogenes. *S. forbessii* contains 79 single copy genes, plus 35 contained within the repeats, including 14 potential pseudogenes. *S. hermonthica* has 76 single copy protein-coding genes, 35 in the repeats and 10 potential pseudogenes. *S. aspera* contains 77 single copy genes, plus 35 in the repeat regions, with 12 potential pseudogenes (Table 2).

**Table 2:** Plastome gene content in Lindenbergia philippensis and eight hemiparasites of the Orobanchaceae

	Total Protein-encoding Genes (Duplicated)	Potential Pseudogenes	tRNA	rRNA
*L. philippensis*	91 (12)	0	30	4
*S. americana*	86 (12)	5	30	4
*P. cheilanthifolia*	87 (8)	0	30	4
*A. virginica*	91 (12)	0	30	4
*T. versicolor*	91 (12)	0	30	4
*B. americana*	111 (17)	9	30	4
*S. forbessii*	149 (70)	14	30	4
*S. hermonthica*	146 (70)	10	30	4
*S. aspera*	147 (70)	12	30	4

The table shows the total number of genes that are predicted to encode proteins, the number of unique coding genes, the number of genes that are potentially pseudogenes, and the number of types of tRNA and rRNA genes. The most leftward column shows the total number of predicted genes, including pseudogenes, while the number in parentheses is the number of these genes that are duplicated because of their presence in the IR. Information sources for this Table are as described for Table 1

### Gene losses

**Fig. 1:**
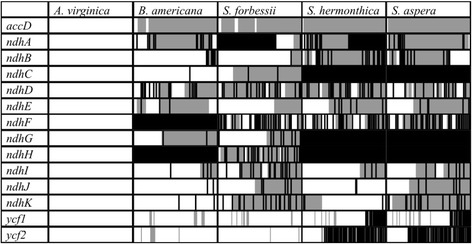
Stop codons and frameshifts in potential pseudogenes. Genes that are missing or predicted as non-functional in at least one of the five species. Solid white bars indicate the gene is present and appears functional in that species, while bars with gray shading or black lines may be pseudogenes. The structure of the protein-encoding portion of the gene is indicated within each box, with the 5′ end being at the far left and the 3′ end at the far right. The size of each box is 100% of the protein-encoding region, so they are not corrected for the different sizes of the coding regions of each gene. Gray shading indicates portions of the genes with a shifted frame, while black lines indicate stop codons. Solid black bars are genes or gene segments that are completely missing from that species. For instance, the figure shows that the 5′ end of *ndhA* is missing in *S. forbessii* and the 3′ end is missing in *S. hermonthica,* but the multiple stop codons and frameshifts in the 5′ end in *S. hermonthica* should guarantee that it is a non-functional gene

In *L. philippensis* and *A. virginica*, *ndhA* is a 2.2 kb gene containing two exons, 539 bp and 553 bp, separated by a 1.1 kb intron. *S. hermonthica* is missing the second exon of *ndhA*, while *S. forbessii* is missing the first exon. *B. americana* and *S. aspera* have both exons, but have a predicted frameshift mutations and premature stop codons in the first exon. All *ndh* genes are present with the structure of functional genes in *A. virginica*. *B. americana* and the three *Striga* species all have one or more stop codons or frameshifts in *ndhB,* starting at amino acid 446 in *B. americana* and 448 in *S. forbessii*. *S. hermonthica* and *S. aspera* both have a frameshift at amino acid 24, suggesting a shared event in their common ancestor, followed by multiple frameshifts and stop codons. *ndhC* is present in *B. americana* but contains a stop codon at amino acid 82. *S. forbessii* contains *ndhC* with a frameshift starting at amino acid 23 followed by multiple stop codons. *ndhC* is missing from both *S. hermonthica* and *S. aspera*. All four species contain multiple frameshifts and stop codons throughout *ndhD* starting at amino acid 36 in *B. americana*, 25 in *S. forbessii*, 4 in *S. hermonthica* and 40 in *S. aspera*. *S. forbessii* contains a stop codon in *ndhE* at amino acid 101. *B. americana* has a stop codon at amino acid 11. *S. hermonthica* has a stop codon at amino acid 40. *B. americana, S. hermonthica,* and *S. aspera* all contain 5′ frameshift mutations, at amino acid 34 in *B. americana* and amino acid 32 in the *Striga* species, but all are independent events. All three species have additional stop codons following the beginning of the frameshift. *ndhF* is missing from *B. americana* and contains a frameshift at amino acid 20 in *S. forbessii* followed by multiple stop codons. *S. hermonthica* and *S. aspera* both contain a stop codon at the third amino acid followed by additional stop codons and frameshifts. *ndhG* contains a frameshift and multiple stop codons starting at amino acid 63 in *B. americana* and amino acid 3 in *S. forbessii*. *ndhG* is missing from *S. hermonthica* and *S. aspera*. *ndhH* is missing from *B. americana*, *S. hermonthica*, and *S. aspera*. *ndhH* is present in *S. forbessii* but contains frameshifts and multiple stop codons starting at amino acid 11. *ndhI* contains a stop codon in *B. americana* at amino acid 90 and in *S. forbessii* at amino acid 17, followed by a frameshift and additional stop codons. *S. hermonthica* contains a frameshift starting at amino acid 47 and *S. aspera* contains a frameshift starting at amino acid 42, both followed by multiple stop codons. *ndhJ* contains stop codons starting at amino acid 140 in *B. americana*. In *S. forbessii, ndhJ* contains a frameshift at amino acid 71 followed by multiple stop codons. *ndhJ* in *S. hermonthica* contains stop codons starting at amino acid 102. *ndhJ* contains a frameshift in *S. aspera* starting at amino acid 44 followed by stop codons. *ndhK* contains a stop codon at amino acid 120 in *B. americana* and amino acid 14 in *S. forbessii*. Both species contain additional stop codons and *S. forbessii* contains a frameshift. *ndhK* in both *S. hermonthica* and *S. aspera* contain a frameshift at amino acid 4 followed by stop codons. Pertinent frameshifts and stop codons are shown in Fig. 1. Table 3 shows mutations shared by two or more species at the identical amino acid that resulted in frameshifts or stop codons.

**Table 3:** List of shared frameshift mutations and stop codons

Gene	Species	Amino Acid	SC or FS
*ndhA*	*B. americana*	19	SC
	*S. hermonthica*	20	SC
	*S. aspera*	20	SC
*ndhB*	*S. hermonthica*	24	FS
	*S. aspera*	24	FS
	*S. hermonthica*	118	SC + FS
	*S. aspera*	118	SC + FS
*ndhD*	*S. hermonthica*	94	FS
	*S. aspera*	94	FS
	*S. hermonthica*	116	FS
	*S. aspera*	116	FS
	*S. hermonthica*	152	FS
	*S. aspera*	152	FS
	*S. forbessii*	269	FS
	*S. hermonthica*	269	FS
	*S. aspera*	269	FS
	*S. hermonthica*	319	FS
	*S. aspera*	317	FS
	*S. hermonthica*	358	FS
	*S. aspera*	356	FS
	*S. hermonthica*	372	FS
	*S. aspera*	370	FS
	*S. hermonthica*	391	FS
	*S. aspera*	389	FS
	*S. hermonthica*	425	FS
	*S. aspera*	424	FS
*ndhF*	*S. hermonthica*	3	SC
	*S. aspera*	3	SC
	*S. forbessii*	20	SC
	*S. hermonthica*	20	SC
	*S. aspera*	20	SC
	*S. hermonthica*	81	FS
	*S. aspera*	91	FS
	*S. forbessii*	617	FS
	*S. aspera*	593	FS
*ndhI*	*B. americana*	90	SC
	*S. forbessii*	89	SC
	*S. hermonthica*	90	SC
	*S. aspera*	90	SC
	*S. hermonthica*	112	FS
	*S. aspera*	112	FS
*ndhK*	*S. hermonthica*	3	FS
	*S. aspera*	3	FS
*accD*	*B. americana*	57	FS
	*S. forbessii*	12	FS
	*S. hermonthica*	12	FS
	*S. aspera*	59	FS
	*B. americana*	92	FS
	*S. forbessii*	47	FS
	*S. hermonthica*	47	FS
	*S. aspera*	129	FS
*ycf1*	*B. americana*	251	FS
	*S. forbessii*	256	FS
	*S. hermonthica*	258	FS
	*S. aspera*	254	FS
	*B. americana*	360	FS
	*S. forbessii*	386	FS
	*S. hermonthica*	392	FS
	*S. aspera*	391	FS
	*S. forbessii*	467	FS
	*S. hermonthica*	491	FS
	*S. aspera*	475	FS
	*B. americana*	674	FS
	*S. forbessii*	648	FS
	*S. hermonthica*	661	FS
	*S. aspera*	660	FS
	*S. hermonthica*	1288	FS
	*S. aspera*	1287	FS
	*S. hermonthica*	1452	SC
	*S. aspera*	1451	SC
	*S. hermonthica*	1462	FS
	*S. aspera*	1461	FS
*ycf2*	*S. hermonthica*	482	FS
	*S. aspera*	489	FS
	*B. americana*	848	FS
	*S. forbessii*	1024	FS
	*S. hermonthica*	987	FS
	*S. aspera*	994	FS

List of frameshift mutations (FS) and stop codons (SC) that are shared by two or more species and the amino acid position in each species. FS and SC in the same box are the result of the identical nucleotide mutation in each species. Stop codons created by frameshift mutations are not included. *S. forbessii* is missing the first exon of *ndhA*, which contains the shared stop codon at the 20th amino acid in the other three species. *ndhE* does not have any shared mutations, but *B. americana, S. hermonthica*, and *S. aspera* have independent insertions at the same aligned amino acid position (35 in *B. americana* and 33 in the two *Striga* species)

*accD* is present in all five species. In *A. virginica*, it appears functional with very few differences in comparison to *L. philippensis*. In the other four species, there are a numerous predicted mutations including multiple frameshifts. However, the 3′ end of this gene is considerably more conserved than the 5′ end of the gene**.** It’s possible that *accD* has a conserved protein domain encoded by the 3′ end of the gene. *ycf1* and *ycf2* also seem to have multiple frameshift mutations in all species, except *ycf2* in *A. virginica*.

### Plastome alignments and phylogenetic analysis

**Fig. 2:**
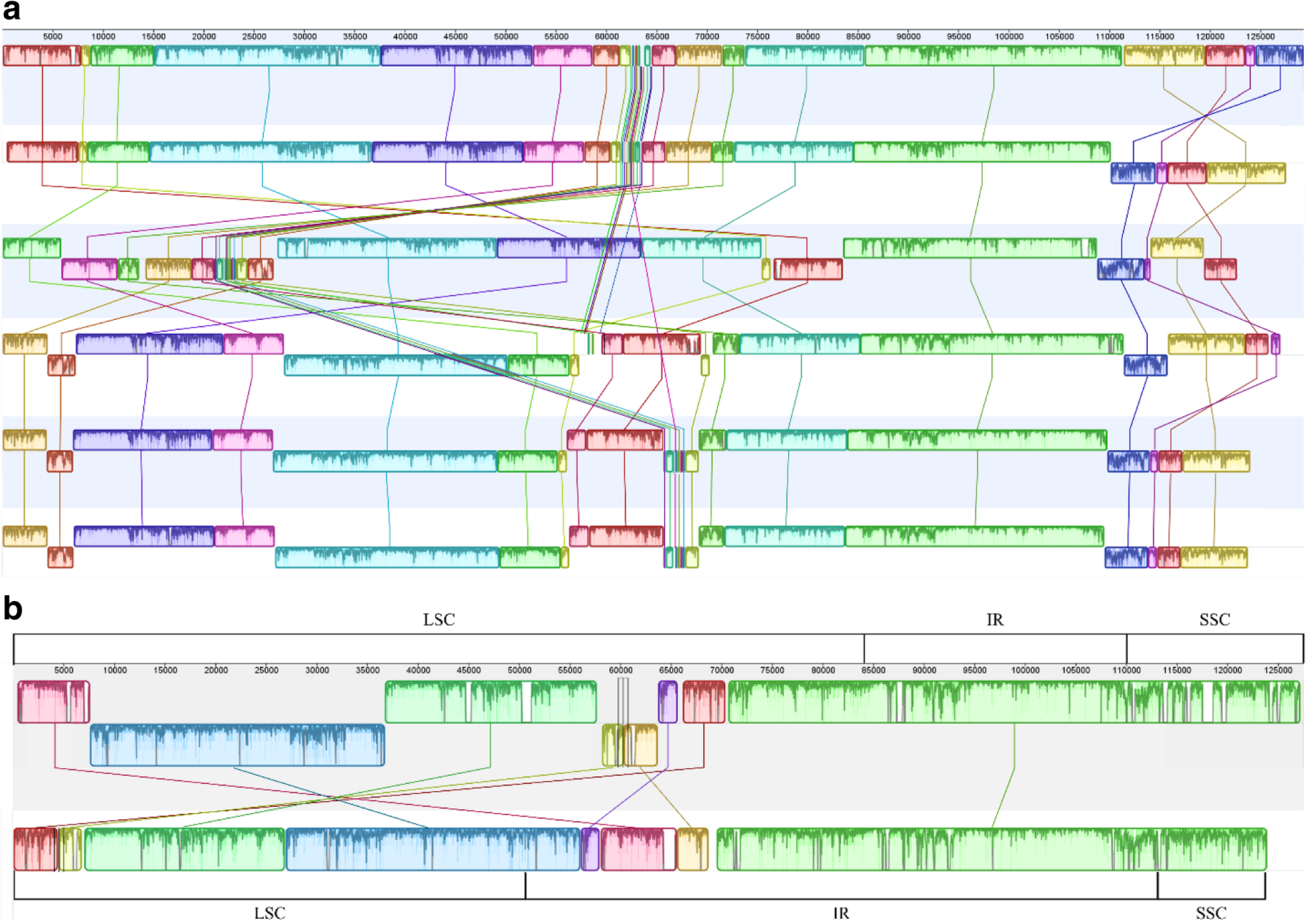
Mauve alignment of plastomes. **a** Alignment done in Mauve 2.3.1 showing plastomes with one copy of the IR taken out. The order of plastomes, from top to bottom, is *L. philippensis*, *A. virginica*, *B.americana*, *S. forbessii*, *S. hermonthica*, and *S. aspera*. Each colored block is a region of collinear sequence among all six plastomes. Blocks on the top row are in the same orientation, while blocks on the bottom row are in inverse orientation. **b** Mauve alignment of *A. virginica* (top) and *S. hermonthica* (bottom). The blocks and colors do not correspond to (**a**). The LSC, IR, and SSC are indicated below each alignment

All six plastomes were aligned with Mauve 2.3.1 and are shown in Fig. 2a. *A. virginica* is largely identical to *L. philippensis*, except that the SSC is in an inverted orientation. All other plastomes contained multiple rearrangements. Within the *Striga* genus, *S. hermonthica* and *S. aspera* have no rearrangements between them, while *S. forbessii* has a single rearrangement within the repeat region. At a higher resolution, Fig. 2b shows the example of the Mauve alignment of just *S. hermonthica* and *A. virginica.*

### The timing of gene loss events

**Fig. 3:**
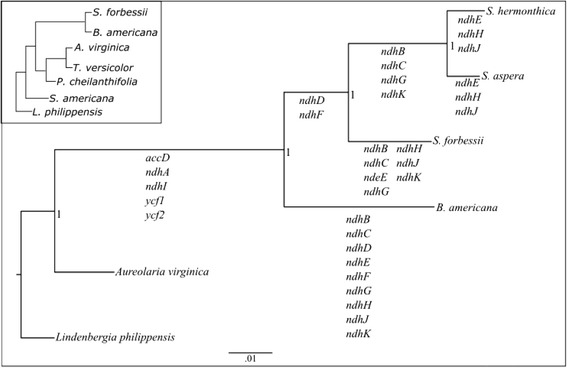
Phylogenetic trees of the studied species and of plastome gene loss. Phylogenetic tree of the investigated hemiparasite plastomes, decorated with the branch of specific inactivational events for the listed genes. These inactivational events included deletions, frameshifts and/or stop codon generation. The inset tree shows the relationships between the species studied in this manuscript to three others that have been previously investigated, *Pedicularis cheilanthifolia*, *Schwalbea americana*, and *Triphysaria versicolor* [19, 20]. The inset tree is from McNeal et al. 2013 [32] and the other tree is from MrBayes (as described in Methods). The branch lengths of the large tree are proportional to the number of substitutions per site with the scale shown at the bottom. The number 1 at each node is the posterior probability, the likelihood that the tree is correct

In many cases, a specific nucleotide location of a sequence change that is predicted to inactivate a gene was shared by more than one species. Hence, it is most likely that such events occurred in a common ancestor of the species that share the event. Because these deletions, frameshifts and/or stop codon creations are predicted to inactivate the gene, then it is appropriate to plot the lineage and relative time of inactivation of each gene onto a phylogenetic tree of the compared species. The phylogenetic tree, with *L. philippensis* as an outgroup, is shown in Fig. 3, with predicted shared gene inactivations indicated as gene names along specific branches.

## Discussion

### Plastome conservation and change

Plastomes perform common and vital functions in virtually all vascular plants, and thus are very powerful tools for phylogenetic analysis. The parasitic plants that are non-photosynthetic lose strong selection for the many plastome genes involved in photosynthesis, but are expected to retain coding capacity for genes that are involved in other essential chloroplast roles. In previous studies of non-photosynthetic plants [13, 14, 18–21], most or all photosynthetic genes are seen to be pseudogenized or lost. This process appears to be caused by random mutation, and is thus thought to be a gradual process that will only be completed at some distant time after the parasitic/non-photosynthetic lifestyle is established. Our observations, akin to those observed by Wicke et al. [20] in a broader but less detailed survey of parasitic plants, indicate that some chloroplast genes are lost even in fully photosynthetic parasites, suggesting that chloroplast roles other than generation of photosynthate may now be provided by the host. Hence, extensive gene loss from the plastome does not link exclusively with a holoparastic lifestyle. If hemiparasitism is a routine step toward the evolution of holoparasitism, then it may be that the plastome gene loss process is as much a cause of holoparasitism as it is an outcome.

Parasitism has evolved independently at least 12 to 13 times within angiosperms [42]. Parasitism within Orobanchaceae is believed to have evolved once and is estimated to have occurred 32–64 mya [43–45]. It is interesting that these different plastomes have come to somewhat different final gene contents and that many sequences are still present despite their loss of apparent function. This suggests that the gene loss process is very slow, especially compared to nuclear DNA loss [46], and raises the possibility that the different adaptations of these different species has led to different plastome gene loss outcomes. For instance, most *ndh* genes were lost early in the lineage leading to the genus *Striga*, but *ndhE*, *ndhH* and *ndhJ* have only been lost in terminal branches, suggesting a possible transitional role for these genes even when the other *ndh* genes were no longer functional. Similarly, *Pedicularis cheilanthifolia* has apparently lost only one plastome gene to pseudogenization, *ndhf*, a gene that has been independently lost in many lineages [28]. All these results confirm the observation that, in hemiparasites, the *ndh* pathway is unnecessary or possibly even selected against. Further analysis of the nature of plastome genome change should provide additional insights into which genetic (and, thus, physiological) functions are lost, in what lineages and at what evolutionary rates and times.

### Plastome size

Most plant plastomes range in size from 120 to 160 kb. *L. philippensis* falls within this range. The *A. virginica* plastome is slightly smaller than that of *L. philippensis*, but still falls within the typical range. The LSC, SSC, and IR of *A. virginica* are all similar in size to those of *L. philippensis* and fall within the general size range for angiosperms. *B. americana* and the three *Striga* species, however, all show interesting size difference in both whole plastome length and the lengths of their LSCs, SSCs, and IRs. All have a larger plastome than *L. philippensis* and above the range generally seen in plants*.* This was unexpected as most parasitic species studied so far have smaller plastome sizes [14, 16, 17, 19–23]. The increase in size can be explained by an increase in the length of the inverted repeats. All five species have an increased inverted repeat size, although *Aureolaria*’s is close to *Lindenbergia* (26,031 bp compared to 25,812 bp) and falls within the typical range of angiosperm inverted repeat size. The other four species have considerably larger repeats above the 20 to 30 kb size generally seen in angiosperms, ranging from 43,864 bp in *Buchnera* to 63,240 bp in *S. forbessii*. Small changes of a few hundred bp in inverted repeat size are common in land plants [47–49], but larger changes as seen here are rare [47–52]. The largest known repeat belongs to *Pelargonium hotorum,* at 75,741 bp [53]. Slight IR expansion has been seen in the parasitic plastomes of *Schwalbea americana* and *Cistanche phelypaea*. However, these expansions were much smaller in size, encompassing 2 genes and 3 genes [19], respectively, compared to the expansions we saw in *Striga* and *B. americana*.

The repeat size increase that we observed is due to an expansion into the single copy regions. All five parasitic species have smaller SSC and LSC regions compared to *L. philippensis*. The *Striga* expansions differ dramatically from the expansion in *B. americana.* In *B. americana*, most of the repeat expansion has been into the SSC region. In the three *Striga* species, there has been some repeat expansion into the SSC region, but most of the expansion has been into the LSC region. There are two similarities in the expansion patterns between the *Striga* species and *Buchnera.* The first is the 8 kb in the LSC region adjacent to the IR (spanning from *trn*H to *psb*K). This region is contained within the repeat of all four species, although the *Striga* IRs extend a further 4.5 kb region into this region. It is possible that the initial 8 kb expansion occurred before the species diverged and was followed by an additional 4.5 kb expansion in the *Striga* lineage. The second similarity is the expansion into the SSC that contains the *ycf*1 gene. The *B. americana* IR includes an additional 0.5 kb containing *rpl*32, suggesting that this was an additional expansion after the lineages diverged. Other than these two similarities, the rest of the repeat expansions are unique to either *B. americana* or the *Striga* species and most likely occurred after the two lineages diverged. These several independent repeat expansions indicate either selection for this phenomenon or a mechanistic anomaly in these lineages that makes this outcome unusually likely, compared to other studied plants. Functionally, these increases in the duplicated regions do not appear to have created any new genes. Because of the process of gene conversion across these recombining repeats [3], it is not expected that any new gene functions would evolve through this IR expansion.

*S. aspera* and *S. hermonthica* show no rearrangements between them or differences between the repeat expansions. There is one major structural difference between the plastomes of these two and that of *S. forbessii*. Within the repeat, a 2.5 kb region containing *cemA* and *petA* is located in a different part of the region in an inverse orientation. One possibility is that repeat expansion occurred independently in the *S. forbessii* lineage and the shared *S. hermonthica/aspera* lineage after the two lineages diverged. The other possibility is that the repeat expansion occurred before the common ancestor of these three species diverged, followed by lineage-specific rearrangement(s) after divergence.

### Missing genes

The NAD(P)H-dehydrogenase complex is involved in one of the multiple pathways that recycles electrons around photosystem I [54, 55, 60]. The NAD(P)H complex functions under stressful conditions and may be essential for photosynthesis under conditions of highly variable light intensities [56, 57]. Eleven subunits are encoded by the plastome: *ndhA, B, C, D, E, F, G, H, I, J,* and *K* [58]. These genes have been lost several times in land plants and are commonly missing in parasitic plants [13, 16, 17, 19, 20, 23, 59–63].

*Ycf*1was the first plastid-encoded protein identified whose presence was shown to be essential for the survival of green plants. *Ycf*1 protein is an essential component of the translocon that is responsible for chloroplast protein transport in green plants [64]. However, no *ycf*1 homolog was detected among Poales, except *Typha latifolia* [65]. In our five studied species, *ycf*1 was observed to be highly divergent, with predicted frameshift mutations. The gene *ycf*2 still has an unknown function [66] and has a functional structure in *A. virginica*, but contains multiple frameshift mutations in *B. americana* and the three *Striga* species. Both *ycf*1 and *ycf*2 have high substitution rates in most of the land plant, including non-parasites, and may have become pseudogenes in some lineages [67, 68]. The 5′ ends of both genes tend to be relatively conserved, while the remaining portions of the genes are more divergent. We observed a similar pattern with the most conserved regions of both genes being in the 5′ end. Complete losses of both *ycf*1 and *ycf*2 have occurred in some monocots [69].

*AccD* encodes the beta subunit of the multimeric acetyl-CoA carboxylase, which mediates the conversion of acetyl-CoA to malonyl-CoA during fatty acid synthesis. The *accD* gene has been lost several times in angiosperms [70], where its function is taken over by nuclear copies [71]. The 3′ region of the *acc*D gene in all five of our studied species is considerably more conserved than the 5′ end. Higher divergence or truncation of the 5′ end of the *acc*D gene also has been observed in several species [19, 20]. We do not have a nuclear genome sequence for any of the species investigated in this manuscript, but we predict that a nuclear *accD* homologue is now functional in the *B. americana* and *Striga* lineages. We also predict that the 3′ end of the *accD* gene may have a separate, plastid-specific, function.

### Plastome alignments

*A. virginica* has no major genic rearrangements compared to *L. philippensis* except an inverted SSC region. The SSC region is frequently flipped in plastomes, with opposite SSC orientations often present in a 50:50 ratio in plant cells [1]. Comparisons between *A. virginica* and *B. americana* are interesting as they are both facultative hemiparasites. Because they are both capable of living without a host, thereby depending heavily on photosynthesis, it is expected that they would have a relatively small number of rearrangements compared to autotrophic plant plastomes. This is the case with *A. virginica.* However, *B. americana* has multiple rearrangements compared to *A. virginica* and *L. philippensis*. The three *Striga* species also have several rearrangements compared to *A. virginica* and *L. philippensis*, as well as compared to *B. americana*. There are no rearrangements between *S. hermonthica* and *S. aspera*, and a single rearrangement between these two and *S. forbessii*. These patterns all fall within an appropriate phylogenetic order that predicts their time and lineage of origin.

The phylogenetic tree agrees with existing phylogenetic analysis as far as the relationship between *L. philippensis*, *A. virginica*, *B. americana*, and the *Striga* genus [72], as well as the relationship of the three *Striga* species to each other with *S. hermonthica* and *S. aspera* being more closely related to each other than to *S. forbessii* [33].

### Plastome instability and parasitic plant function

Although all five of the plant species investigated in this study are obligate photosynthetic organisms, plastome instability was found to be extensive in four species. These results are in agreement with previous observations for other parasitic plants [13–21], and has also been seen more rarely in non-parasites [29, 45, 53, 65, 73–79]. Hence, plastome instability is a source of genomic variation that provides the raw material for natural selection. In any green plant, this selection is expected to lead to overall conservation of gene content, with exceptions for any chloroplast function that can be provided by the environment directly. For parasitic plants, the host plant is a primary environmental contributor, potentially for photosynthate and many of the numerous small molecules that are generated by pathways localized to the chloroplast. The *ndh* pathway is lost in many lineages, suggesting that its role in photosynthesis is often dispensable. Given the rapid and near-complete loss of this pathway independently in so many lineages, it suggests that *ndh* may actually be selected against in some plant lineages. Future studies that return this pathway to a species from which it has been naturally lost would provide an excellent opportunity to investigate the lineages and conditions under which the *ndh* gene array might benefit or debilitate a plant.

In this same vein, all of the gene changes observed in this study could be investigated for their role in plant fitness. Expression levels of the genes that differ in their copy number across these taxa, for instance due to their inclusion or lack of inclusion in the IRs, could suggest a role for the generation and retention of such rearrangements across many plant lineages. Natural variation for plastome structure should be tested across more Orobanchaceae species, both to acquire better ideas of the lineages and rates of such rearrangements, but also to provide the raw material for comparisons of the functional outcomes of these rearrangements. With the ability to transform chloroplasts now available in many plant species [80–83], investigations of natural plastome variation and its role in organismal function could be directly compared to experiments that test these gene effects in engineered transgenics.

## Conclusions

We sequenced and assembled the chloroplast genomes from five species of hemiparasitic plants from the Orobanchaceae. We compared the assemblies to the available plastome assembly from *Lindenbergia philippensis*, an autotroph from the Orobanchaceae. *Aureolaria virginica* was almost identical to *L. philippensis* in terms of plastome structure, gene content, and gene order. *B. americana, S. forbessii, S. hermonthica*, and *S. aspera* all showed a high number of rearrangements and both physical and functional gene loss. The most interesting result was the increase in the plastome size in these four species, since most parasites have plastomes with decreased sizes. The increase was due to the repeat region expanding into the single copy regions, although what effect this has on the plastome is not yet known. *B. americana, S. hermonthica*, and *S. aspera* all had missing *ndh* genes, while these three species and additionally *S. forbessii* had multiple *ndh* genes that are potentially nonfunctional due to predicted frameshift mutations and stop codons. No other genes were missing from any of the species, but *accD, ycf1,* and *ycf2* all had high levels of divergence with frameshifts in *B. americana, S. forbessii, S. hermonthica,* and *S. aspera*, as well as stop codons in *ycf1* and *ycf2* in *S. hermonthica* and *S. aspera*.
